# QM/MM Description of Newly Selected Catalytic Bioscavengers Against Organophosphorus Compounds Revealed Reactivation Stimulus Mediated by Histidine Residue in the Acyl-Binding Loop

**DOI:** 10.3389/fphar.2018.00834

**Published:** 2018-08-03

**Authors:** Alexander Zlobin, Yuliana Mokrushina, Stanislav Terekhov, Arthur Zalevsky, Tatiana Bobik, Anastasiya Stepanova, Maria Aliseychik, Olga Kartseva, Sergey Panteleev, Andrey Golovin, Alexey Belogurov, Alexander Gabibov, Ivan Smirnov

**Affiliations:** ^1^Faculty of Bioengineering and Bioinformatics, Lomonosov Moscow State University, Moscow, Russia; ^2^Shemyakin-Ovchinnikov Institute of Bioorganic Chemistry, Russian Academy of Sciences, Moscow, Russia; ^3^Institute of Fundamental Medicine and Biology, Kazan Federal University, Kazan, Russia; ^4^Institute of Molecular Medicine, Sechenov First Moscow State Medical University, Moscow, Russia

**Keywords:** ultrahigh-throughput screening, butyrylcholinesterase, bioscavenger, organophosphorus compound, computer design, paraoxon

## Abstract

Butyrylcholinesterase (BChE) is considered as an efficient stoichiometric antidote against organophosphorus (OP) poisons. Recently we utilized combination of calculations and ultrahigh-throughput screening (uHTS) to select BChE variants capable of catalytic destruction of OP pesticide paraoxon. The purpose of this study was to elucidate the molecular mechanism underlying enzymatic hydrolysis of paraoxon by BChE variants using hybrid quantum mechanical/molecular mechanical (QM/MM) calculations. Detailed analysis of accomplished QM/MM runs revealed that histidine residues introduced into the acyl-binding loop are always located in close proximity with aspartate residue at position 70. Histidine residue acts as general base thus leading to attacking water molecule activation and subsequent SN2 inline hydrolysis resulting in BChE reactivation. This combination resembles canonical catalytic triad found in active centers of various proteases. Carboxyl group activates histidine residue by altering its p*K*_a_, which in turn promotes the activation of water molecule in terms of its nucleophilicity. Observed re-protonation of catalytic serine residue at position 198 from histidine residue at position 438 recovers initial configuration of the enzyme’s active center, facilitating next catalytic cycle. We therefore suggest that utilization of uHTS platform in combination with deciphering of molecular mechanisms by QM/MM calculations may significantly improve our knowledge of enzyme function, propose new strategies for enzyme design and open new horizons in generation of catalytic bioscavengers against OP poisons.

## Introduction

*De novo* protein design based on methods of structural bioinformatics is an actively developing field of modern life science. New biocatalysts created with this approach may be used for various purposes including therapeutic intervention (Vellard, 2003). Given natural limitations on the number of recombinant enzymes’ variants that can be tested experimentally, computational methods provide unique opportunities to narrow polypeptide sequence space to a set with reasonable size to be evaluated in search for novel properties, such as: fitting of new amino acid sequences into the existing structures (Dahiyat and Mayo, 1997; Socolich et al., 2005); creation of new proteins with previously unknown topology (Kuhlman et al., 2003; Koga et al., 2012); generation of enzymes with altered substrate specificity (Chen et al., 2009); design of enzymes with completely new catalytic activity (Zanghellini et al., 2006; Siegel et al., 2010); optimization of enzymes’ binding affinity (Lippow et al., 2007; Tinberg et al., 2013); creation of artificial proteins capable of selective paired interaction (Grigoryan et al., 2009); improvement of enzyme thermostability (Korkegian et al., 2005) or immunogenicity (Osipovitch et al., 2012; Salvat et al., 2014). Computational *de novo* design of enzymes catalyzing chemical reactions for which there are no natural analogous (Jiang et al., 2008; Rothlisberger et al., 2008; Siegel et al., 2010) may be regarded as breakthrough opening new era in enzymology. Usage of computational methods can be applied for generation of virtual libraries of enzyme’s active center (Smirnov et al., 2016) or as a starting point for the subsequent rational design of new biocatalysts with improved activity toward substrate of interest (Zheng et al., 2014).

Butyrylcholinesterase (BChE), a highly abundant serum enzyme, is a suicidal inactivator of organophosphorus toxins (OP). The exact function of BChE is unclear, nevertheless it is regarded as potent natural antidote against acetylcholine esterase poisons (Ilyushin et al., 2013; Lockridge, 2015; Terekhov et al., 2015). Since BChE is required to be administered in stoichiometric or even larger amounts in comparison with OP, application of BChE as an antidote toward OP poisoning may be restricted. It is evident that variants of BChE capable of catalytic destruction of OP are highly demanded (Aharoni et al., 2004; Valiyaveettil et al., 2011). BChE double mutant G117H/E197Q showed both reactivation and slowing down of aging process. However, example of its practical application for the treatment of OP poisoning clearly demonstrated that OP binding was also significantly decreased. This way the inhibition of endogenous BChE and AChE by OP will occur much earlier than interception of OP by bioscavenger, and therefore no protective effect would be achieved (Malisi et al., 2009). In other words, the development of effective catalytic antidotes is possible only if they either have highly efficient binding (similar to the wild-type BChE) or have extremely effective catalysis (*k*_2_/*K*_M_≈10^7^ M^-1^min^-1^). That was demonstrated for mutant forms of paraoxonase and phosphotriesterase (Jbilo et al., 1994; Mesulam et al., 2002), which had a protective effect against 2 × LD_50_ cyclosarin (GF) and VX when the enzyme was administered at a dose of only 0.2 and 2 mg/kg, respectively. All these findings clearly show the need for further development of design approaches to create new catalytic bioscavengers. Recently we utilized combination of calculations and high-throughput screening technologies in order to create new BChE variants capable for catalytic degradation OP pesticide paraoxon (Terekhov et al., 2017). However, precise reason for this emergence of reactivation ability is unknown. The purpose of this study is to determine the molecular mechanism of paraoxonase activity mediated by newly described BChE variants using hybrid quantum mechanical/molecular mechanical (QM/MM) calculations.

## Materials and Methods

### Recombinant BChE Variants Expression and Purification

Genomic DNA from clones 14 and 15 was extracted as described previously (Lõoke et al., 2011) and used as a PCR template with primer pair a/b: (a) GCTTACTCGGAAGATGACATCATAATTGCAACAA (b) CTCCTTCGATCGACGCATGCAGAAAGCTCTGG. The product was cloned into the pFUSE-BChE-6xHis plasmid using *NheI* and *NcoI* restriction sites. These plasmids were used for transfection of HEK-293F cells using 293fectin Transfection Reagent (Thermo Fisher Scientific) according manufactures’ recommendations. Expression of BChE variants was carried out in FreeStyle 293-F Cells media (Thermo Fisher Scientific) in 250 ml culture flasks for 5 days. The cultural media was collected, concentrated using 30 kDa cut-off filter (Millipore) and further purified using TALON Metal Affinity Resin (Clontech) according manufactures’ recommendations.

### Enzyme Activity Assay

Bimolecular inhibition constant (*k*_1_/*K*_i_) of BChE variants was determined according to the Kitz–Wilson method (Kitz and Wilson, 1962). The residual BChE activity was determined according to the Ellman’s method (Ellman et al., 1961). The activity was measured using Varioscan Flash Multimode Reader (Thermo Fisher scientific) at λ_abs_ = 405 nm. All data was derived from three technical replicates.

### Cluster Analysis

To generate conformation states of BChE variants we employed Rosetta loopmodel (Stein and Kortemme, 2013) approach and generated 100 structures for each variant using PDB ID 1XLW as a template, where DEP was removed due to software limitations (Nachon et al., 2005). To reduce dimension of possible conformers we clustered conformations with AffinityPropagation (Frey and Dueck, 2007; Reshetnikov et al., 2018) according to the position of histidine(s) residue(s) relative to the catalytic serine residue: (i) histidine(s) residue(s) should be directed toward catalytic serine residue; (ii) amino acid residues in acyl-binding loop do not overlap with DEP (after structural alignment with 1XLW). Due to the tautomeric nature of histidine residue initial structures were generated with both possible protonated forms. DEP residue was copied from 1XLW into every analyzed structure.

### QM/MM Calculations

The molecular mechanics (MM) approach was applied to the MM part of the system using parameters from the parm99sb-ildn force field with corrections (Lindorff-Larsen et al., 2010). The quantum mechanics (QM) subsystem was described utilizing DFTB approach (Grimme et al., 2010, 2011; Gaus et al., 2012, 2013). Side-chain atoms of catalytic triad (Ser^198^, His^438^, and Glu^325^), Glu^197^, DEP, newly introduced histidine residues from acyl-binding loop and neighboring water molecules in radius of 5 Å from DEP and histidine residues were included in the QM part of the system. Each simulation system was filled with water molecules according to the TIP3P model, and the total charge was neutralized with Na^+^ or Cl^-^ ions. Water and ions were equilibrated around the protein-DEP complexes by carrying out a 10-ps MD simulation with freezed position of protein-DEP conjugate, 50-ps with restrained position and 1-ns without restraints. For each cluster 10 independent replicas of preparation runs were carried and then analyzed to pick only productive conformations (with any water molecule in direct access to DEP, which was assumed as attacking) for subsequent reaction. Prepared systems were subjected to the QM/MM simulation with the GROMACS/DFTB package (Abraham et al., 2015; Kubar et al., 2015). The time step was set to 0.2 fs. Temperature coupling with Velocity Rescale (Bussi et al., 2007) scheme allowed observation of the behavior of systems at 300K. The total length of each initial ranking simulation was set to 10 ps. A metadynamics approach was used to overcome the activation barrier (Laio and Parrinello, 2002). In simulation run we used one composite collective variables (CV): distance Og(Ser^198^)–P(DEP) minus distance P(DEP)–Ow(attacking water). A Gaussian history-dependent repulsive potential with a width of 0.006 nm and height of 2 kJ/mol was added every 40 steps. Additional upper wall restraints with kappa constant of 3000 were put on values of d(Og–P) and d(P–Ow) higher than 2.7 Å (minimal distance observed in QM/MM equilibration runs) to keep system within reaction conditions. To simulate specifically in-line hydrolysis a restraint with kappa constant of 500 was applied on angle Og–P–Ow being 180°. PLUMED plugin was used to apply metadynamics and restraints (Tribello et al., 2014). In total 320 QM/MM metadynamics runs were performed.

After initial ranking thorough energy profile scan was carried on selected systems. For this we utilized state-of-the-art TTMetaD enhanced sampling approach tailored to specifically address calculations of chemical reactions (Dama et al., 2014; Sun et al., 2016, 2017). To achieve better performance QM subsystem was redefined to exclude unnecessary water molecules that do not participate in proton transfer. Asp^70^ was added since it proved to be crucial during initial ranking simulations. We used the same CV as stated before with potential width of 0.0045 nm and height of 1 kJ/mol. TTMetaD bias factor was set to be 42 kT, and initial guess for transition states (TS) coordinates was derived from ranking round to be -0.04 nm and 0.04 nm for first and second TS, respectively. Each profile was explored in parallel by 28 separate walkers to produce cumulative sampling time around 400 ps which corresponds to 2 million steps. A set of additional potentials was imposed to qualify for reaction mechanism devised from ranking round and could be accessed in Supplemental Information. Trajectories were analyzed with python scripts with the help of MDAnalysis (Michaud-Agrawal et al., 2011), and snapshots of trajectories with minimal energy barriers were created with PyMOL package.

Higher-level QM single-point energy calculations were performed using PBE0 (Perdew et al., 1996; Adamo and Barone, 1998). To further improve accuracy of calculations we utilized D3BJ set of empirical corrections (Grimme et al., 2010, 2011). To speedup calculation we limited basis set to def2/TZVP (Weigend and Ahlrichs, 2005) basis set with excluded f orbitals and enabled RIJCOSX approximation (Kossmann and Neese, 2010).

## Results and Discussion

### Selection of BChE Variants Capable of Catalytic Inactivation of Paraoxon Utilizing Ultrahigh-Throughput Screening

**FIGURE 1:**
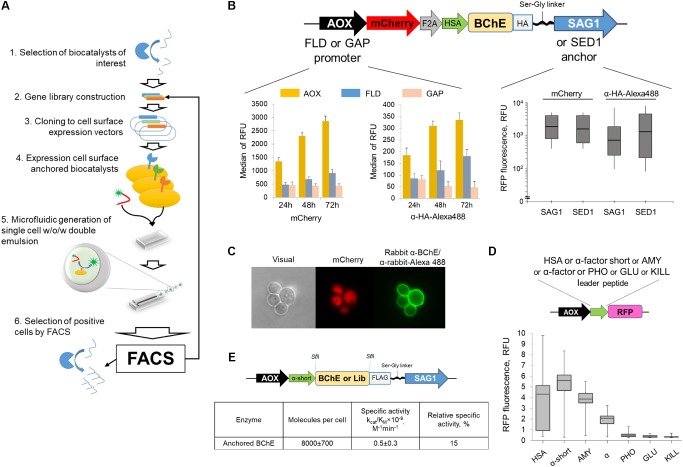
**(A)** The scheme of the universal ultrahigh-throughput screening (uHTS) platform for biocatalysts selection. For the selected biocatalyst (1) the gene library is constructed (2). The library is further cloned into the specialized vectors (3) and transformed into the *Pichia pastoris* yeast cells, that present biocatalysts on the cell surface (4). The cells with displayed biocatalysts are screened by uHTS platform: (5) single cells are compartmentalized in the aqueous droplets of a double w/o/w emulsion with the fluorogenic substrate utilizing microfluidic technology. (6) Droplets with cells expressing active enzyme and therefore containing the fluorescent product are sorted by FACS. **(B)** Optimization of the plasmid vector for cloning of the BChE library for expression of the enzyme on the yeast cell surface. Scheme of the genetic constructs for expression of enzyme exposed on the cell surface (top). Comparative analysis of the surface-displayed BChE expressed under control of different promoters (left panel) and amount of BChE displayed on the cell surface promoted by different polypeptide anchors (right panel). **(C)** Extracellular release of the RFP, fused with different signal peptides. The genetic construct is presented on top. **(D)** Staining of yeast cells transformed by genetic construct (shown on top) by polyclonal anti-BChE antibodies (green fluorescence). Red fluorescence corresponds to the intracellular expression of the mCherry. **(E)** The scheme of the optimized genetic construct coding for the BChE variants used for uHTS (top). Analysis of enzymatic activity of surface-displayed WT BChE by Ellman’s assay.

BChE variants were selected by previously developed ultrahigh-throughput screening technology of individual clones with different functional activity based on the usage of microfluidic droplets of the double water-oil-water emulsion (Terekhov et al., 2017). Schematically this method is described on **Figure 1A**. The advantage of applied methodology consists in generation of a monodisperse emulsion, therefore all individual droplets have the same dimensions. In this case, the fluorescence signal generated by the individual drops depends only on the concentration of the encapsulated fluorescent product, which in turn is formed as a result of the enzyme reaction, occurred in the drop. In order to provide appropriate quantification of the enzyme activity, it is necessary to achieve its effective and reproducible expression on the cell surface. Therefore, the optimization of the genetic construct encoded BChE, anchored on the surface of the yeast cells, in terms of the promoter, the leader peptide and the anchor sequence was carried out (**Figures 1B,D**).

The initial plasmid vector contained DNA coding for red fluorescent protein (RFP) mCherry fused with HA-tagged BChE via “self-processing” F2A peptide (Yang S. et al., 2008). This construct provides simultaneous production of a reporter and enzyme using a single mRNA transcript. Inducible aldehyde oxidase 1 (AOX1) and formaldehyde dehydrogenase 1 (FLD1) promoters or constitutive glyceraldehydes-3-phosphate dehydrogenase (GAP) promoter were used to determine the effect of the promoter on the level of production of the biocatalyst on the surface of the yeast cell (**Figure 1B**, left panel). Analysis of intracellular and membrane-allocated fluorescence revealed that the most efficient expression was achieved in the case of AOX1 promoter.

The effect of the anchor sequence on the expression of the recombinant biocatalyst on the surface of the yeast cell was studied using plasmids that differ only in the anchoring gene: either containing the sequence of the agglutinin surface antigen 1 (SAG1) subunit or the C-terminal fragment of the sperm-egg adhesion 1 (SED1) protein. After 72 h of analytical expression, the cells were stained with green fluorescent antibodies to HA and analyzed by cytofluorometry (**Figure 1B**, right panel). Despite in the case of SED1 anchor sequence the amount of the enzyme was much higher, the SAG1 provided the narrow distribution of enzyme on the surface, which is of the fundamental importance for the creation of cell-based libraries. The minimum possible distribution of the amount of marshaled enzyme is necessary for the subsequent correct selection of improved biocatalysts from libraries, the choice of biocatalysts from a wide distribution can lead to the selection of molecules without improved properties, but simply from better producing cells. Thus, for the creation and further selection of libraries by the yeast cell display method, it is more expedient to use SAG1. SED1 can be used to obtain clones-producers with the maximum level of protein production (in the medium or on the surface of the cell). Finally, the genetic construct used for the BChE yeast display contained methanol-inducible promoter AOX1, sequences of the red fluorescent reporter protein mCherry, F2A peptide, HSA leader to transport the expression product to the culture medium, the gene sequence of the enzyme to be studied, the HA epitope for immunofluorescent detection of the anchored enzyme, and the SAG1 sequence is linked serine glycine linker, which provides the attachment of the enzyme to the surface of the yeast cell (**Figure 1C**).

In order to determine the efficiency of the leader peptides providing the secretion of the protein of interest to the external medium, the α and α-short, HSA, KILL, AMY, PHO, and GLU signal sequences (please refer Supplementary Methods for details) were implemented in the signal reading frame with the nucleotide sequence coding for RFP. After 72 h of analytical expression of 96 clones of each leader peptide variant, the cells were removed by centrifugation and the fluorescent signal in the supernatants was analyzed (**Figure 1D**). Leader peptides α-short and AMY provide a high level of release of the RFP, whereas HSA demonstrated variation in the level of release of the RFP by different clones. Final genetic construct for library generation contained FLAG-tagged BChE gene with α-short leader peptide and C-terminal SAG1 sequence linked through serine glycine linker under control of AOX1 promoter. Transformation of yeast cells by this plasmid resulted in the appearance of a fully functional protein on the surface of the yeast cell. The analysis of the number of anchored molecules and calculation of the specific activity of the enzyme revealed that the developed yeast cellular display allows to obtain around 8000 molecules on a surface with specific activity that equals to the 15% of the activity of BChE in solution (**Figure 1E**).

The resulting vector was used to create a cell-based library of BChE variants. An acyl-binding loop ^284^TPLSV^288^ was used for random mutagenesis. Two variants of BChE exhibiting resistance to inactivation by paraoxon were selected (Terekhov et al., 2017). We further compared activity of the selected variants and 10 randomly picked clones from the initial library against parental substrate butyrylthiocholine and paraoxon (**Table 1**). Our data suggested that BChE clones containing sequences ^284^HTIHG^288^ (clone 14) and ^284^PSHSG^288^ (clone 15) hydrolyzed paraoxon, whereas clone 14 retained a significant level of specific activity toward butyrylthiocholine. Before only one known BChE substitution G117H was known to grant reactivation ability (Lockridge et al., 1997). Deciphering the precise molecular mechanism of newly found reactivating variants can provide valuable insight into this emerged organophosphate activity and help to devise new strategies for design of catalytic bioscavengers against OP poisons.

**Table 1:** Kinetic and structural analysis of specific esterase and paraoxonase activity of selected BChE variants.

Enzyme		Relative BChE activity, %	Activity against paraoxon		284-288 acyl-binding loop sequence
			*k*_1_/*K*_i_ × 10^-4^, M^-1^min^-1^	*k*_2_ × 10^3^, min^-1^	
BChE	WT	100.0	17 ± 2	–	Thr-Pro-Leu-Ser-Val
BChE variants from library without screening	1	32.8	–	–	Pro-Thr-Leu-Arg-Gly
	2	22.4	–	–	Pro-Gln-Ile-Ser-Ser
	3	54.6	–	–	Pro-Pro-Leu-Arg-Ser
	4	19.4	–	–	Pro-Pro-Ile-Asn-Gly
	5	5.2	–	–	Ser-Thr-Ile-Ser-Gln
	6	12.2	–	–	Pro-Pro-Lys-Asn-Gln
	7	1.4	–	–	Lys-Arg-Asn-Arg-Val
	8	1.4	–	–	Thr-Arg-Leu-Arg-Ile
	9	1.1	–	–	Asn-Thr-Lys-Lys-Glu
	10	2.0	–	–	Asn-Lys-Ser-Asn-Val
BChE variants selected against paraoxon	clone 14	39.0	16 ± 2	6 ± 0.6	**His**-Thr-Ile-**His**-Gly
	clone 15	8.0	1.5 ± 0.2	7 ± 0.6	Pro-Ser-**His**-Ser-Gly

### Computational Analysis of Molecular Mechanism of Paraoxon Hydrolysis by BChE Variants

**FIGURE 2:**
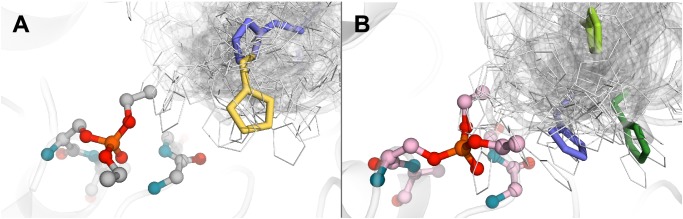
Positions of histidine residue selected after calculation and clusterization of structures corresponding to the clone 14 **(A)** and clone 15 **(B)**.

Presence of proline residue in initial acyl-binding loop in principle should resulted in distinct and rigid configuration of this loop. Substitution of proline residue may lead to the increased loop flexibility, which in turn should be accounted during modeling process. In order to address this issue we generated 100 possible loop conformations for selected clones 14 and 15. Computational analysis of every possible loop configuration would require enormous computational resources, thus we were forced to decrease quantity of initial models. Therefore, we performed cluster analysis of generated configurations and finally obtained 11 clusters for both BChE variants (**Figure 2**). To further reduce number of initial models we withdraw configurations in which side chain of histidine residues do not fit into the reaction hemisphere. Despite both mutants are capable for reactivation, their activity is significantly decreased in comparison with common enzymes. That in turn means that frequency of catalytic events, especially reactivation with water, is very rare. Absence of relevant data on whether one or two water molecules and which one of two histidine residues in clone 14 are involved in the catalysis brought additional difficulties in the modeling. We therefore utilized a two-step approach: first we performed a “wide” round of multiple independent and relatively short calculation runs to get insight on principle reaction paths and rank all starting systems. Then only for the best of them we performed “deep” round of calculations aimed at accurate reconstruction of energy landscape. For both rounds we utilized metadynamics technique to simplify overcoming of energetic barriers since, on the one hand, it does not require *a priori* knowledge of reaction mechanism and, on the other hand, gives high-quality free energy estimate if mechanism is specified (Sun et al., 2017). Combination of metadynamics with hybrid QM/MM simulation recently had been shown as a suitable instrument for studying of slow enzymatic reactions involving organophosphorus compounds (Smirnov et al., 2016).

**FIGURE 3:**
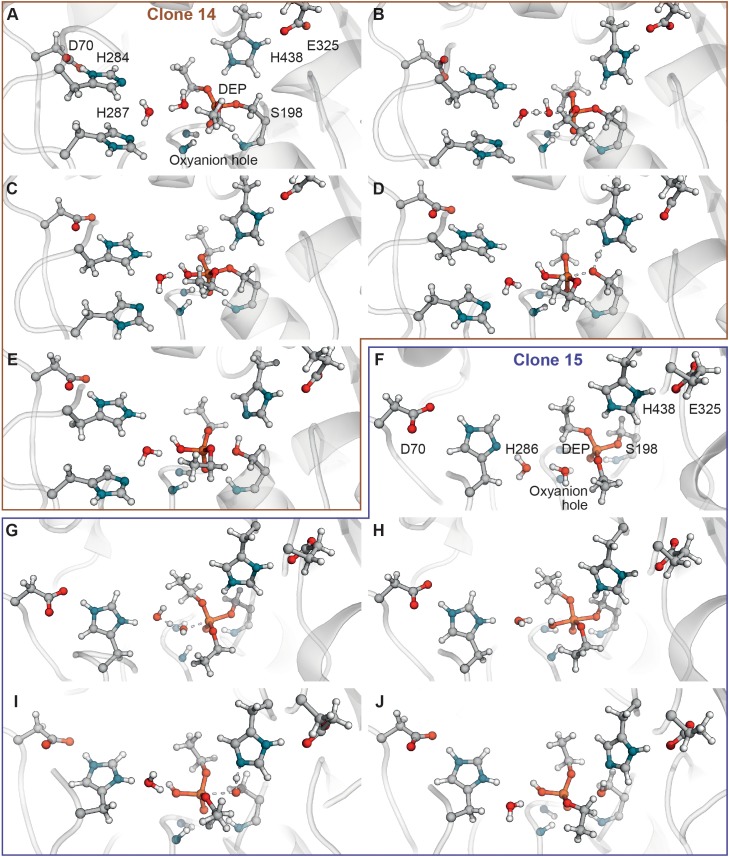
Computer analysis of the catalytic mechanism of paraoxon hydrolysis by selected BChE variants. **(A–E)** Snapshots of the reactivation reaction path of paraoxon hydrolysis by clone 14. **(F–J)** Snapshots of the reactivation reaction path of paraoxon hydrolysis by clone 15.

Analysis of ranking round trajectories with the lowest energy barriers for both BChE variants revealed that histidine residue coordinate attacking water molecule therefore acting as a general base (**Figures 3B,G**). We showed that two histidine residues in acyl-binding loop of clone 14 participate in reaction of paraoxon hydrolysis in completely different ways. Histidine residue at position 284 always acts as a general base, whereas His^287^ never does this and instead coordinates water molecule (**Figures 3A–E**). Protonation state of histidine residue acting as general base evidently is crucial for the reaction. The most successful runs for clone 14 with ranking barrier of 32–33 kcal/mol were observed in cluster 11 containing His^284^ with proton on the δ-nitrogen atom (**Table 2**). Alternative clusters or protonation states yielded much higher energetic barriers or totally disrupted the reaction. In case of clone 15 with the 30 kcal/mol ranking barrier of the beneficial configuration originated from cluster 11 with proton on the ε-nitrogen atom (**Figures 3F–J** and **Table 3**). In both clones reaction ends with the proton transfer from His^438^ to Ser^198^ thus completely restoring the enzyme’s initial state (**Figures 3E,J**). Detailed analysis of the accomplished runs revealed that histidine residues are always located adjacent to the aspartate residue at position 70. This combination resembles canonical pair of histidine and either aspartate or glutamate residues in the catalytic core of different proteases (Buller and Townsend, 2013). Carboxyl group functions as an activator of histidine residue by altering its p*K*_a_ and therefore promoting deprotonation of corresponding nucleophile (**Figures 3B,G**). Similar positioning of histidine residue and activating amino acid was recently proposed as a rational strategy for design of BChE variants capable of reactivation (Lushchekina et al., 2018).

**Table 2:** Results of QM/MM ranking analysis for BChE clone 14.

Cluster	Protonation	Estimated energy barrier, kcal/mol	Molecular observations
5	284HID	39.61	His^284^ acts as general base but does not interact with Asp^70^
5	284HIE	–	Thr^285^ restricts access of water molecule to phosphorus atom
7	284HID	41.30	bbbHis^284^ is positioned too far and does not act as general base
7	284HIE	42.23	
11	284HID-287HID	32.14	His^284^ acts as general base and interacts with Asp^70^
11	284HID-287HIE	32.91	His^284^ acts as general base and interacts with Asp^70^
11	284HIE-287HIE	–	Lack of positioning of water molecule for attack
11	284HIE-287HID	–	

*HIE: proton on the ε-nitrogen atom, HID: proton on the δ-nitrogen atom. For clusters 5 and 7 His^*287*^ does not positioned towards active side residues and thus its protonation states were neglected*.

**Table 3:** Results of QM/MM ranking analysis for BChE clone 15.

Cluster	Protonation	Estimated energy barrier, kcal/mol	Molecular observations
1	286HID	44.62	His^286^ acts as general base but does not interact with Asp^70^
1	286HIE	41.76	
10	286HID	–	His^286^ restricts access of water molecule to phosphorus atom
10	286HIE	–	
11	286HID	39.10	His^286^ acts as general base but does not interact with Asp^70^
11	286HIE	30.22	His^286^ acts as general base and interacts with Asp^70^

*HIE: proton on the ε-nitrogen atom, HID: proton on the δ-nitrogen atom*.

**FIGURE 4:**
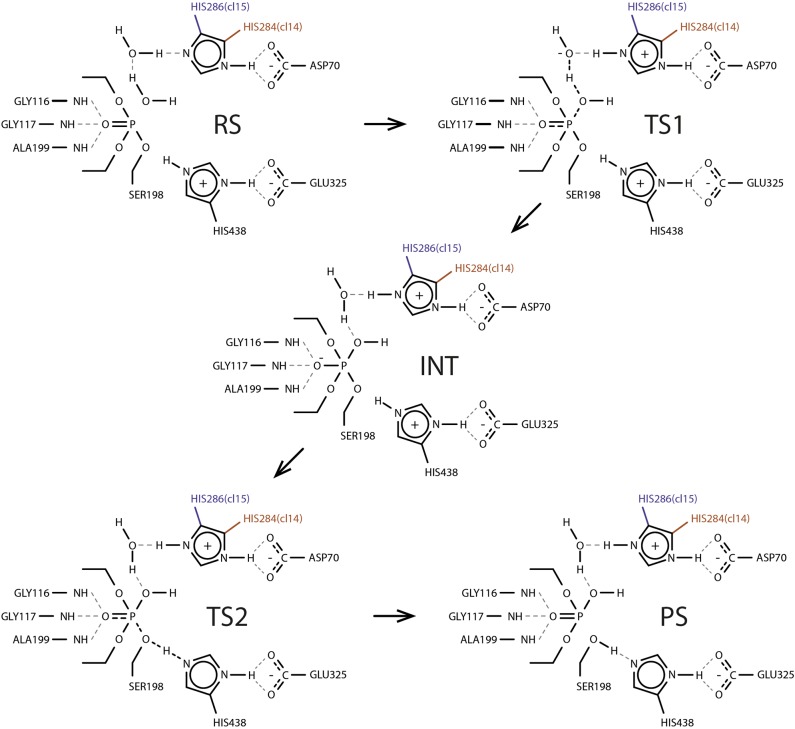
Proposed generalized reaction mechanism of paraoxon hydrolysis by clones 14 and 15 based on trajectory snapshots. RS, reactant state; TS1, first transition state; INT, intermediate; TS2, second transition state; PS, product state.

**FIGURE 5:**
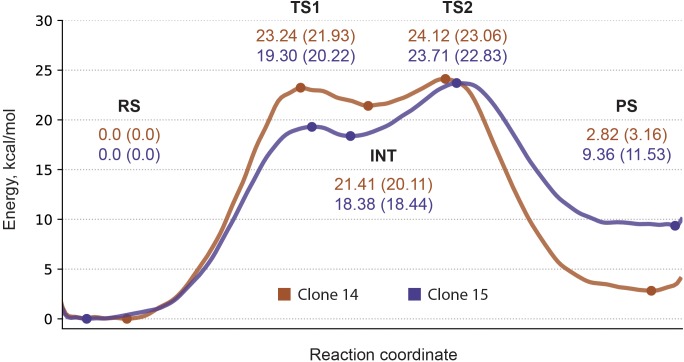
Energy profile for clones 14 and 15. Metadynamics profile shown in solid line. Reported are energy values for reaction states with PBE0 calculations shown in parentheses.

With all the valuable insight gained from ranking calculation round we note that energy barrier estimates carry only qualitative and relative value which originates mainly from the choice of CV—all proton transfer events are not explicitly treated as a part it—together with insufficient sampling time. Therefore for two best systems—cluster 11 of clone 14 with both histidines as δ-protonated and cluster 11 of clone 15 with ε-protonated histidine—we further reconstructed energy profiles in the second calculation round with dramatically increased sampling time. Obtained profiles clearly show five distinct states: reagents state, first transition state, intermediate, second transition state, and products state (**Figure 4**). Estimated energy barriers—24.12 and 23.71 kcal/mol for clone 14 and 15, respectively—are in good agreement with values of 23.06 and 22.96 kcal/mol calculated from experimental data using Eyring-Polanyi equation (**Figure 5**).

Worth to mention that while DFTB approach was already used to study enzymatic reaction profiles and reproduced structural features well (Gruden et al., 2017), the overall performance in energy profile reconstruction based on single point energy evaluation for stationary points was reported to be below desirable level (Kulakova et al., 2015; Vasilevskaya et al., 2016). However, it was mentioned that the lack of accounting for configurational entropy may cause significant errors in the free energy surface for enzymatic reactions (Yang Y. et al., 2008; Hayashi et al., 2012). Therefore we utilized DFTB in the context of TTMetaD calculations, which as a valuable addition provides free energy profiles of the entire reaction path and not just stationary points. On the other hand, since DFTB geometries are regarded as reliable (Gruden et al., 2017), we decided to further validate our findings by performing energy calculations with higher-level hybrid functional PBE0 which has proven to be both accurate giving typical error less than 2 kcal/mol and robust for different type of systems (Luo et al., 2011). Energy profile reconstructed this way on points derived from metadynamics calculations produced similar values for energy estimate—23.06 and 22.83 kcal/mol for clone 14 and 15, respectively—thus also demonstrating good agreement with the experiment and supporting the choice of our methods.

Summarizing, active center of combinatorially selected BChE variants mediates canonical SN2 inline hydrolysis of covalent complex with paraoxon by incorporating additional nucleophile-base-acid triad as an outcome of introduced mutations. Observed reprotonation of catalytic Ser^198^ by proton transfer from His^438^ recovers initial state of the enzyme, ready for the next catalytic turn. Data reported herein suggest that BChE with multiple substitutions in principle may seize paraoxonase activity and gives new ideas on design strategies to finally produce an efficient organophosphate enzyme. Synergy of the uHTS platform and *in silico* QM/MM explanation and prediction of amino acid residues’ catalytic function may open new horizons in generation of bioscavengers against OP poisons including but not limited to paraoxon molecule.

## Author Contributions

AlZ, ArZ, and AnG performed the QM/MM calculations. YM and TB designed the genetic construct. ST designed and performed the screening and analyzed the kinetic experiments. AS and MA performed the molecular cloning. OK and SP performed the kinetic experiments. AB wrote the paper. AlG and IS contributed to conception and design of the study and wrote the paper.

## Conflict of Interest Statement

The authors declare that the research was conducted in the absence of any commercial or financial relationships that could be construed as a potential conflict of interest.
