# Supplementary material for: QM/MM Description of Newly Selected Catalytic Bioscavengers Against Organophosphorus Compounds Revealed Reactivation Stimulus Mediated by Histidine Residue in the Acyl-Binding Loop
Source: Front Pharmacol. 2018 Aug 3;9:834. doi: 10.3389/fphar.2018.00834 (PMC6085465; doi:10.3389/fphar.2018.00834)
Supplement: Supplementary file 1 [file Presentation_1.PDF]

Supplemental information

QM/MM description of newly selected catalytic bioscavengers against organophosphorus compounds revealed reactivation stimulus mediated by histidine residue in the acyl-binding loop

by Zlobin et al.

Supplemental Information includes Supplementary Methods and References 1-3

Supplementary Methods

Yeast display of BChE. Recombinant human butyrylcholinesterase (BChE) was produced in *Pichia pastoris* GS115 (Invitrogen) using expression vector pPICZ-mCherry-F2A-HSAss-AfeI/PvuI-HA-SAG1 based on the pPICZαA (Invitrogen) (Terekhov et al., 2017). The fragment encoding BChE was PCR-amplified from pFUSE PRAD-F2A-BChE (Terekhov et al., 2015) and cloned into the vector pPICZ-mCherry-F2A-HSAss-AfeI/PvuI-HA-SAG1 digested with *AfeI* and *PvuI* resulting in pPICZ/AOX-Enz-SAG1. The plasmid vectors pPICZ/FLD-Enz-SAG1 and pPICZ/GAP-Enz-SAG1 with strong inducible promoter FLD1 (formaldehyde dehydrogenase 1 promoter) and constitutive promoter GAP (glyceraldehyde-3-phosphate dehydrogenase promoter) were generated by cloning of PCR-amplified sequence of pFLD and pGAP from *Pichia pastoris* genomic DNA into vector pPICZ/AOX-Enz-SAG1 using *BglII* and *MfeI* restriction sites. The plasmid vector pPICZ/AOX-Enz-SED1 was designed by cloning of PCR-amplified sequence of SED1 anchoring region from *Saccharomyces cerevisiae* genomic DNA into vector pPICZ/AOX-Enz-SAG1 using *XbaI* and *SalI* restriction sites. The seven different signal sequences, namely serum albumin from *Homo sapiens* (HSA), α-mating factor signal peptide from *Saccharomyces cerevisiae* (α-short), α-amylase from *Aspergillus niger* (AMY), α-mating-factor prepropeptide from *Saccharomyces cerevisiae* (α), acid phosphatase PHO1 from *Pichia pastoris* (PHO), glucoaminase from *Aspergillus awamori* (GLU) and killer protein from *Saccharomyces cerevisiae* (KILL) were fused with RFP as reporter gene. The coding sequence of RFP was amplified from pTagRFP-C (Evrogen) using specific primers and cloned utilizing *EcoRI/Acc65I* sites into the pGAPZαA vector (Invitrogen) to generate the pGAPZαA/RFP with α-factor signal sequence. Constructs with other six signal peptide were obtained by annealing of corresponding primer pairs and replacing α-factor signal sequence from pGAPZαA/RFP utulizing *BstBI* and *EcoRI* restriction sites resulting in pPICZ/GAP-Leader-RFP. The plasmid vector pPic9k-α-BChE-FLAG-anchor was generated by cloning of BChE gene PCR-amplified from pFUSE PRAD-F2A-BChE (Terekhov et al., 2015) into expression vector pPic9k-α-short-SfiI-FLAG-SAG1 using *SfiI* site. This vector pPIC9k-based (Invitrogen) vector contained the AOX1 promoter, α-short leader peptide and SAG1 anchor sequences. The degenerated primers:

[a] GCGCTAGCTGCGGCCCAGCCGGCCGAAGATGACATCATAATTGCAACAAAGAA
[b] CTTTGTAGTCCATCAGGCCCCCGAGGCCGAGACCCACACAACTTTCTTTCTTG
[c] CCCATAGGGGACAACAAATGCTTC
[d] AACTTTGGTCCGACCGTGGATG
[e] GAAGCATTTGTTGTCCCCTATGGGMVWMVWMDWMRWVDWAACTTTGGTCCGACCGTGGATG

were used to amplify regions flanking the mutated 284-TPLSV-288 loop of WT human BChE and cloned into expression vector pPic9k-α-BChE-FLAG-anchor using *SfiI*. Approximate diversity of the library was estimated as 9×10^5 individual representatives. All vectors were linearized by *PmeI* and transformed into *Pichia pastoris* GS115 as previously described (Wu and Letchworth, 2004).

Cell staining. Staining was performed using antibodies to the BChE and antibodies against HA-epitope conjugated with Alexa 488. Cells were loaded into a cell sorter FACS AriaIII (BD) and visualized using the inverted fluorescence microscope Eclipse Ti (Nikon) with standard FITC and Texas Red filters.

Screening of library of BChE variants. The screening procedure is described in (Terekhov et al., 2017). Yeasts producing a library of anchored BChE variants were grown overnight in liquid culture using YPD medium and induced by growth in BMMY medium. Subsequently, the yeasts were washed twice with 50 mM potassium phosphate buffer pH 7.4 and resuspended in 50 mM potassium phosphate buffer pH 7.4. Next, a cell suspension was incubated with OPs – 0.2 mM paraoxon (POX, Sigma-Aldrich) for 0.5 hour at 25°C, washed with 50 mM potassium phosphate buffer pH 7.4 supplemented with 2 μM of the paraoxon, filtered using 20 μm solvent filter A-313

(IDEX), and encapsulated with BChE substrate, including 100 µM butyrylthiocholine and 100 µM 3-(7-Hydroxy-2-oxo-2H-chromen-3-ylcarbamoyl)acrylic acid methyl ester. After 3 hours droplets with highest fluorescence were selected using FACS. The regenerated yeast colonies were analyzed for BChE activity in a 384-well plate. The most active clones after OP inactivation and ten randomly picked clones from unscreened library were selected and sequenced to determine substitutions in the 284-TPLSV-288 sequence of WT human BChE.

Plumed setup for TTMetaD calculation

```
# Plumed setup file with placeholders
# $resname.resnum.atomname$

### Main variables ###

dOP: DISTANCE ATOMS=$SDP.198.OG$,$SDP.198.P$
dPO: DISTANCE ATOMS=$SDP.198.P$,$SOL.530.OW$
aOPO: ANGLE ATOMS=$SDP.198.OG$,$SDP.198.P$,$SOL.530.OW$

PROT1: COORDINATION GROUPA=$SOL.530.OW$,$SOL.531.OW$,$HIE.286.ND1$
GROUPB=$SOL.530.HW1$,$SOL.530.HW2$,$SOL.531.HW1$,$SOL.531.HW2$ R_0=0.1 D_0=0.03
PROT2: COORDINATION GROUPA=$SDP.198.OG$,$HIP.438.NE2$ GROUPB=$HIP.438.HE2$ R_0=0.1 D_0=0.03

dWH: DISTANCE ATOMS=$SOL.531.OW$,$HIE.286.ND1$
dWW: DISTANCE ATOMS=$SOL.530.OW$,$SOL.531.OW$
dHS: DISTANCE ATOMS=$SDP.198.OG$,$HIP.438.NE2$
dHE: DISTANCE ATOMS=$ASP.70.OD2$,$HIE.286.NE2$
aHhE: ANGLE ATOMS=$ASP.70.OD2$,$HIE.286.HE2$,$HIE.286.NE2$

### Metadynamics ###

CV: COMBINE ARG=PO,OP COEFFICIENTS=-1,1 POWERS=1,1 PERIODIC=NO
mCV: METAD ARG=CV HEIGHT=1.0 SIGMA=0.0045 GRID_MIN=-0.225 GRID_MAX=0.225 GRID_BIN=300 PACE=40
FILE=MHILLS WALKERS_N=28 WALKERS_ID=0 WALKERS_DIR=../walkers TRANSITIONWELL0=-0.04
TRANSITIONWELL1=0.04 TTBIASFACTOR=42

### Potentials ###

aOPO_lw: LOWER_WALLS ARG=aOPO AT=2.5 KAPPA=10000.0
CV_uw: UPPER_WALLS ARG=CV AT=0.15 KAPPA=10000.0
CV_lw: LOWER_WALLS ARG=CV AT=-0.15 KAPPA=10000.0

PROT1_lw: LOWER_WALLS ARG=PROT1 AT=3.5 KAPPA=10000.0
PROT2_lw: LOWER_WALLS ARG=PROT2 AT=0.7 KAPPA=10000.0

dWH_uw: UPPER_WALLS ARG=dWH AT=0.3 KAPPA=10000.0
dWW_uw: UPPER_WALLS ARG=dWW AT=0.3 KAPPA=10000.0
dHS_uw: UPPER_WALLS ARG=dHS AT=0.35 KAPPA=10000.0
dHE_uw: UPPER_WALLS ARG=dHE AT=0.35 KAPPA=10000.0
aHhE_lw: LOWER_WALLS ARG=aHhE AT=2.5 KAPPA=10000.0

### Writeout ###

FLUSH STRIDE=50
PRINT ARG=dOP,dPO,aOPO,aOPO_lw.bias,CV,mCV.bias STRIDE=10 FILE=CV
PRINT ARG=PROT1,PROT1_lw.bias,PROT2,PROT2_lw.bias STRIDE=50 FILE=PROT
PRINT ARG=dWH,dWH_uw.bias,dWW,dWW_uw.bias,dHS,dHS_uw.bias,dHE,dHE_uw.bias,aHhE,aHhE_lw.bias
STRIDE=50 FILE=GEOM
```

Supplementary references

Terekhov, S., Smirnov, I., Bobik, T., Shamborant, O., Zenkova, M., Chernolovskaya, E., Gladkikh, D., Murashev, A., Dyachenko, I., Palikov, V., Palikova, Y., Knorre, V., Belogurov, A., Jr., Ponomarenko, N., Blackburn, G.M., Masson, P., and Gabibov, A. (2015). A novel expression cassette delivers efficient production of exclusively tetrameric human butyrylcholinesterase with improved pharmacokinetics for protection against organophosphate poisoning. *Biochimie* 118**,** 51-59.

Terekhov, S.S., Smirnov, I.V., Stepanova, A.V., Bobik, T.V., Mokrushina, Y.A., Ponomarenko, N.A., Belogurov, A.A., Jr., Rubtsova, M.P., Kartseva, O.V., Gomzikova, M.O., Moskovtsev, A.A., Bukatin, A.S., Dubina, M.V., Kostryukova, E.S., Babenko, V.V., Vakhitova, M.T., Manolov, A.I., Malakhova, M.V., Kornienko, M.A., Tyakht, A.V., Vanyushkina, A.A., Ilina, E.N., Masson, P., Gabibov, A.G., and Altman, S. (2017). Microfluidic droplet platform for ultrahigh-throughput single-cell screening of biodiversity. *Proc Natl Acad Sci U S A* 114**,** 2550-2555.

Wu, S., and Letchworth, G.J. (2004). High efficiency transformation by electroporation of Pichia pastoris pretreated with lithium acetate and dithiothreitol. *Biotechniques* 36**,** 152-154.
